# A Manœuvre in Abdominal Surgery

**Published:** 1897-06-12

**Authors:** 


### A MANŒUVRE IN ABDOMINAL SURGERY.

What is to be done when, after the removal of gall stones from the gall bladder, the incision in that viscus cannot be brought up to the abdominal wall? By most surgeons it has not been considered safe to suture the gall bladder and close the abdomen, although this course has been adopted in a fair number of instances. When, then, the incision in the gall bladder cannot be fixed to the abdominal opening the operator may either pack and drain, or contrive a passage out of omentum, as described by Mayo Robson, or use the tube invented by Murphy for the purpose, or, as has been advised by Mayo Robson, he may tuck in the parietal peritoneum, and suture it to the margins of the incision in the gall bladder.

Mr. Morton, of Bristol, has gone still further in this direction. He has peeled the peritoneum off the inner surface of the abdominal wall, so as to obtain sufficient to tuck it down, and unite it to the edges of the incision in the gall bladder, even when deeply placed. By this manœuvre the peritoneal cavity is shut off, and a sort of open tube is constructed leading from the gall bladder to the surface.

Mr. Morton suggests that the same method might be adopted with advantage in dealing with a pelvic or deep-seated abdominal abscess before making an incision into it.

